# Genome-Wide Identification, Evolution and Expression Profile Analysis of NAC Transcription Factor in *Simmondsia chinensis*

**DOI:** 10.3390/cimb45070344

**Published:** 2023-06-29

**Authors:** Fan Xia, Xiaoyu Liang, Lina Tan, Wen Sun, Xiaogang Dai, Hanwei Yan

**Affiliations:** 1 Laboratory of Modern Biotechnology, School of Forestry and Landscape Architecture, Anhui Agricultural University, Hefei 230036, China; xiafan3483@163.com (F.X.); ljxnyq6675@sina.com (X.L.); 13965947503@163.com (L.T.); 18895426856@163.com (W.S.); 2 Key Laboratory of Tree Breeding & Germplasm Improvement, Southern Modern Forestry Collaborative Innovation Center, College of Forestry, Nanjing Forestry University, Nanjing 210037, China; xgdai@njfu.edu.cn

**Keywords:** NAC, jojoba, identification, evolution, expression

## Abstract

NAC transcription factors (TFs) are one of the largest plant-specific gene families and play important roles in plant growth, development, and the biotic and abiotic stress response. Although the sequencing of Jojoba (*Simmondsia chinensis*) has been completed, the genome-wide identification and analysis of its NAC TFs has not been reported. In this study, a total of 57 genes were identified in Jojoba, which were divided into eight groups based on phylogenetic analysis. The genes clustered in the same groups have a similar gene structure and motif distribution. Based on the analysis of *cis*-elements in NAC TFs, nine *cis*-acting elements were identified in the promoter region that involved in light response, hormonal response, and stress response. Synteny analysis showed a greater collinearity between Jojoba and *V. vinifera* than *Arabidopsis thaliana*. The 24 genes in the Jojoba NAC TFs are derived from fragment replication, which may be the main source of NAC amplification. Gene expression analysis identified seven genes that were highly expressed in seeds. The differential expression analysis of NAC TFs in cotyledon and embryonic axis tissues showed that the expression of 10 genes was up-regulated and 1 gene was down-regulated. This study provides more information on the classification, gene structure, conserved motif, and evolution of NAC TFs in Jojoba, facilitating further exploration of their specific functional analysis in Jojoba seed development.

## 1. Introduction

Transcription factors (TFs) are a class of proteins that specifically interact with *cis*-acting elements in the gene promoter region of eukaryotes [1]. NAC TFs are one of the largest families of TFs widely present in plant species [2], which are named according to the NAM (no apical meristem) found in *Petunia hybrida* [3], the ATAF1/2, and CUC2 (cup-shaped cotyledon) found in *Arabidopsis thaliana* [4]. The N-terminal contains a conserved NAC domain consisting of about 150 amino acids, and the C-terminal is a highly transcriptional regulatory region that is a common feature of NAC proteins [5].

Currently, the genome-wide identification of NAC TFs in some plants has been completed, for example, 117 NAC genes in *Arabidopsis thaliana* [6], 163 genes in *Populus trichocarpa* [7], 125 NAC genes in *Phyllostachys edulis* [8], 269 genes in *Glycine max* [9], and 83 genes in *Sesamum indicum* [10]. Meanwhile, a variety of regulatory functions of NAC in plant physiology have also been revealed, including drought response [11], cold response [12], salt stress [13], lateral root development [14], leaf senescence [15], embryo development [16], and the biosynthesis of secondary metabolites [17]. OsNAC2 was overexpressed in Oryza sativa to promote root growth by regulating auxin and cytokinin [18]. The overexpression of ANAC019, ANAC055, and ANAC072 in Arabidopsis transgenic plants improves tolerance to drought [19]. In *Arabidopsis thaliana*, overexpressed ANAC087 accelerates the process of leaf senescence [20]. Additionally, NAC TFs play a vital role in the development of plant seeds. VvNAC26 in *Vitis vinifera* regulates seed development by influencing ABA and ethylene hormone synthesis pathways [21]. OsNAC20 and OsNAC26 have essential functions in the formation and storage of starch and protein in rice seeds [22]. Similarly, TaNAC19 in wheat (*Triticum aestivum* L.) also plays a key role in the cumulative mechanism of seed storage protein (SSP), a protein that determines the quality of its grains [23]. TaNAC019 enables the expression of the SSP accumulation mechanism by directly activating high molecular weight glutenin and indirectly regulating the expression of TaSPA, an ortholog of maize *Opaque2*.

Jojoba (*Simmondsia chinensis*) is a woody plant native to southern North America and is now widely cultivated around the world. Jojoba has a high economic value, and the liquid wax ester extracted from its seeds as a raw material has positive application prospects in cosmetics, medicine, food, and other industries [24]. Jojoba genome sequencing was published in 2020 [25]. So far, the systematic analysis of the NAC gene family in Jojoba has not been reported. Therefore, the comprehensive analysis and expression patterns of NAC TFs could lay a theoretical basis for the regulatory mechanism of wax ester production in jojoba seeds.

In this study, 57 NAC genes in Jojoba were identified based on the completed sequencing of genome data. We further analyzed their characteristics, including multiple sequence alignment, gene structure, and promoter analysis. Information on the phylogenetic relationship, Ka/Ks ratio, and collinearity was obtained for evolutionary analysis. Importantly, the expression data on different stages of seeds was exploited using RNA-seq. These results may provide a basis for further validation of the relevant function of the NAC gene during the development of Jojoba seeds.

## 2. Materials and Methods

### 2.1. ScNAC TF Database Searches

We downloaded the Jojoba genomic data from the Beijing Institute of Genomics (BIG) data center (http://bigd.big.ac.cn/gwh, accessed on 26 November 2022) by using accession no. GWHAASQ00000000, and applied it to the NAC TFs identification process [25]. Subsequently, 96 Arabidopsis NAC protein sequences were downloaded from Arabidopsis Information Resource site (https://www.arabidopsis.org/browse/genefamily/index.jsp, accessed on 26 November 2022). Then the genes in Jojoba genomic data with homologous sequences to the Arabidopsis NAC members were identified using local BLASTP [26] (E value cutoff of 1 × 10^−5^), and these genes were considered as candidates for the Jojoba NAC TFs. Pfam is a collection of multiple alignments and profile hidden Markov models of protein domain families [27]. To verify that all candidate genes belonged to the NAC family, a hidden Markov model of Pfam was used to check whether they had a specific NAM domain (PF02365). The protein sequences of all NAC candidate members were submitted to the Pfam website and their domains were identified by an hmmscan search, HMMER (http://www.ebi.ac.uk/Tools/hmmer/search/hmmscan, accessed on 26 November 2022), with all parameters as default. The redundant sequences and sequences without a NAM domain were removed from the dataset. The identified NAC genes were renamed based on their location on the Jojoba chromosome. Information about the NAC family of Jojoba genes, including coding sequence (CDS), isoelectric point (PI), and molecular weight (MW) information, was obtained by the Expasy—ProtParam tool. Maplnspect (http://www.plantbreeding.wur.nl/uk/software_mapinspect.html, accessed on 26 November 2022) was used to draw the chromosomal location of the ScNAC genes.

### 2.2. Phylogenetic Analysis

Multiple sequence alignments between the amino acids of the NAC family were performed by MEGA7.0 (version 7.0.21) software (http://www.megasoftware.net/, accessed on 3 December 2022), which is set as the default parameter. A phylogenetic tree was constructed using the neighbor-joining with 1000 bootstrap replicates on MEGA 7.0 [28]. BLASTP [26] (E value cutoff of 1 × 10^−5^) was used to perform similarity searching for ScNACs and NAC members in *Arabidopsis thaliana*. Based on the phylogenetic analysis of the NAC members in Jojoba and *Arabidopsis thaliana* as well as sequence similarity, homologs of each ScNAC in *Arabidopsis thaliana* were identified (Table S1).

### 2.3. Analysis of Exon/Intron Structure, Conserved Motifs, and Promoter Element

The exon/intron gene structure of each ScNAC was visualized based on the Gene Structure Display Server (http://gsds.cbi.pku.edu.cn, accessed on 10 December 2022) [29], with all parameters as default. Additionally, the amino acid sequences of this family were submitted to the online MEME motif search tool, using a maximum pattern number of 20 and default parameters [30]. The *cis*-acting elements in promoter were identified by PlantCARE (http://bioinformatics.psb.ugent.be/webtools/plantcare/html/, accessed on 3 December 2022) [31], which are located in the 2000-bp region upstream of each gene transcription start site. The *cis*-acting elements involved in the same response process were given the same named label. Visualization was conducted through TBtools’ Simple Biosequence Viewer, with all parameters as default.

### 2.4. Syntenic Analysis and Ka/Ks Ratios

The whole genomes of *Arabidopsis thaliana* (TAIR 10) and *Vitis vinifera* (IGGP_12x) were downloaded from TAIR [32] and the Ensemble genome [33] database, respectively. The duplication of the NAC gene was identified based on MCScanX [34] (http://chibba.pgml.uga.edu/mcscan2/, accessed on 3 December 2022). First, the Jojoba protein sequences were aligned to each other and the protein sequences from Arabidopsis and grape using BLASTP, with an E-value cutoff of 1 × 10^−10^. Then, under the MCScanX default parameter settings, we identified the synteny regions. Finally, the results were visualized using Circos [35].

Gene pairs were identified with BLASTN. The synonymous substitution rate (Ks), non-synonymous substitution rate (Ka) and Ka/Ks ratio between the homologous gene pairs were obtained by KaKs_Calculator2.0 [36]. The calculation of the divergence time of the NAC gene pair is based on the formula T = Ks/2λ × 10^−6^ Mya (λ = 7.0 × 10^−9^).

### 2.5. Expression Analysis of ScNAC in Different Tissues and Stages of Developing Seed

The gene expression data of ScNAC in different tissues and developmental stages of Jojoba seed were obtained based on published RNA-seq data in Jojoba [25]. The RNA-seq data can be obtained from the NCBI GEO database (accession no. GSE130603) In this experiment, seeds were divided into three developmental stages based on their average weight, early (0.2 g), middle (0.45 g), and late (0.7 g), and the average weight of seeds used for tissue-specific RNA-seq experiments was 0.45 g. Each tissue contained five biological replicates, each consisting of three seeds. The expression data from different tissues and the developmental stages of seeds were visualized by pheatmap (CRAN—Package pheatmap (hppt://cran.r-project.org/web/packages/pheatmap/index.html), accessed on 12 March 2023).

### 2.6. Differential Expression Analysis of ScNAC in Different Tissues of Developing Seed

Based on the same RNA-seq data as above, the differential expression data of the cotyledon and embryonic axis tissues were obtained [25]. The average seed weight of these tissues was 0.45 g, which was in the mid- to mid-late stage of oil accumulation. The differential expression of ScNAC genes in the cotyledon and embryonic axis tissues was analyzed using DESeq2 with default settings [37]. The difference significance was calculated using more stringent q value than *p* value, in that they also account for the false discovery rate rather than the false-positive rate [38]. The genes with an absolute log_2_FC (log of fold change) value less than 1 were filtered. A volcano plot was created using the Jojoba differential expression genes in the cotyledons and embryonic axis, and the ScNAC genes with an absolute log_2_FC value greater than 2 were annotated on the volcano plot. The volcano plot was obtained by using TBtools’ Volcano Plot with default parameters.

## 3. Results

### 3.1. Identification of NAC TFs in Jojoba

A total of 57 genes were identified, all of which contained a NAM domain (PF02365) specific to a family of NAC TFs. According to their location on the chromosome, they were named ScNAC1 to ScNAC57. The coding sequence (CDS) of the NAC TFs ranged from 814 bp (ScNAC22) to 13877 bp (ScNAC15) in length. Their molecular weights (MWs) ranged from 8.75 kDa (ScNAC22) to 75.33 kDa (ScNAC40), with an average of 39.22 kDa. The isoelectric point (PI) varied from 4.34 (ScNAC22) to 9.84 (ScNAC10), and the average was 6.88. Of these, 29 members had isoelectric points less than 7 and 28 had isoelectric points greater than 7, suggesting that they may function under different physiological conditions [39]. In addition, Jojoba has fewer NAC members than *Vitis vinifera* (74 members) [40] and *Arabidopsis thaliana* (117 members) [6], which may be due to the fact that it does not undergo additional genome duplications in addition to the whole-genome triplication shared among all eudicots [25].

### 3.2. Phylogenetic Analysis of NAC TFs

**Figure 1:**
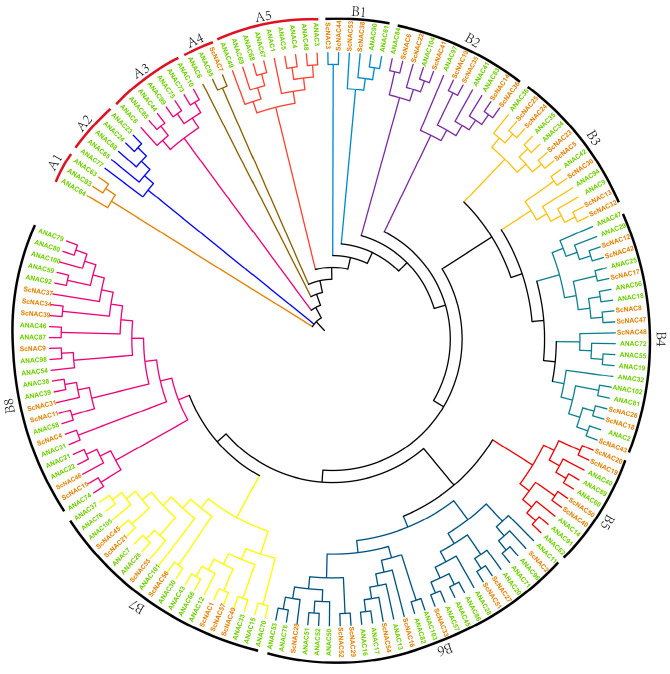
Phylogenetic tree of NAC TFs in *Simmondsia chinensis* and *Arabidopsis thaliana*. The tree was built by the neighbor-joining (NJ) in MEGA7. Different subgroups A1~A5, B1~B8, are shown in different colors. *Simmondsia chinensis* and *Arabidopsis thaliana* are shown in brown and green, respectively.

In order to investigate the phylogenetic relationships of ScNAC TFs, a phylogenetic tree was constructed by neighbor-joining (NJ) using 57 proteins from Jojoba and members of the NAC family of *Arabidopsis thaliana* (Table S1). As shown in Figure 1, NAC TFs were divided into two major groups, A and B, based on a previous study about the classification of NAC domains in *Oryza sativa* and *Arabidopsis thaliana* [6]. Groups A and B had five and eight subgroups, respectively. There was only one ScNAC gene in group A and 56 ScNAC genes in group B. The maximum number of ScNACs in the subgroup is nine (B4, B6, B8) and the minimum number is one (A4) (Figure 1).

Moreover, previous studies of NAC TFs suggested that genes clustered together in the evolutionary tree may have similar functions [41]. Notably, regulatory genes related to seed dormancy and germination emerged in B5 subgroup, such as ANAC060 (AT3G44290.1), ANAC040 (AT2G273000.1), and ANAC089 (AT5G22290.1) [42]. The members in the B5 subgroup may have a relevant function during seed development (Table S3).

### 3.3. Gene Structure and Motif Composition Analysis

**Figure 2:**
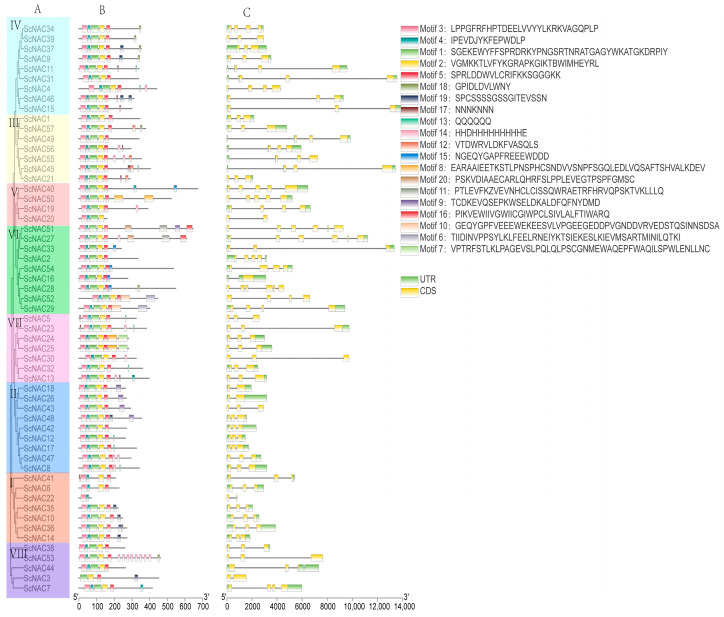
Phylogenetic relationships, conserved motifs, and gene structure of ScNAC genes. (**A**) Phylogenetic tree of Jojoba protein sequences constructed by neighbor-joining (NJ) in MEGA7, with 1000 bootstrap replicates. NAC members in Jojoba were classified into groups I to VIII based on the results of *Arabidopsis* subgroup classification, with different groups represented by different colors. (**B**) Distribution of the conserved motif predicted by MEME in the NAC protein sequence. The sequence of motif 1 to motif 20 was shown in the upper right corner. (**C**) NAC gene structure, yellow for exons, gray lines for introns, green for untranslated regions (UTR).

To explore the evolutionary relationships among ScNACs, a phylogenetic tree was constructed using 57 protein sequences from Jojoba. Based on the phylogenetic analysis of NAC members in Jojoba and *Arabidopsis (*Figure 1), we divided the NAC members in Jojoba into groups I~VIII. From group I to VIII, its members are 7, 9, 7, 9, 4, 9, 7, 5, respectively. In total, 20 conserved motifs were predicted through the MEME program to explore the structural diversity and similarity of NAC TFs in Jojoba. Most NAC members contained five motifs (motif 3, motif 4, motif 1, motif 2, and motif 5), which are components of the highly conserved N-terminus of NAC. It is noteworthy that certain motifs occur in certain groups, such as motif 11 in group VI and motif 7 in group VII (Figure 2). This may indicate that these particular motifs have specific functions.

At the same time, we compared the exon/intron structure in Jojoba. The number of introns varied from 1 to 5. Most ScNAC genes contained two introns. Six genes contained one intron, thirteen genes contained three introns, three genes contained four introns, and three genes contained four introns (Figure 2).

### 3.4. Analysis of Cis-Elements in NAC TFs

**Figure 3:**
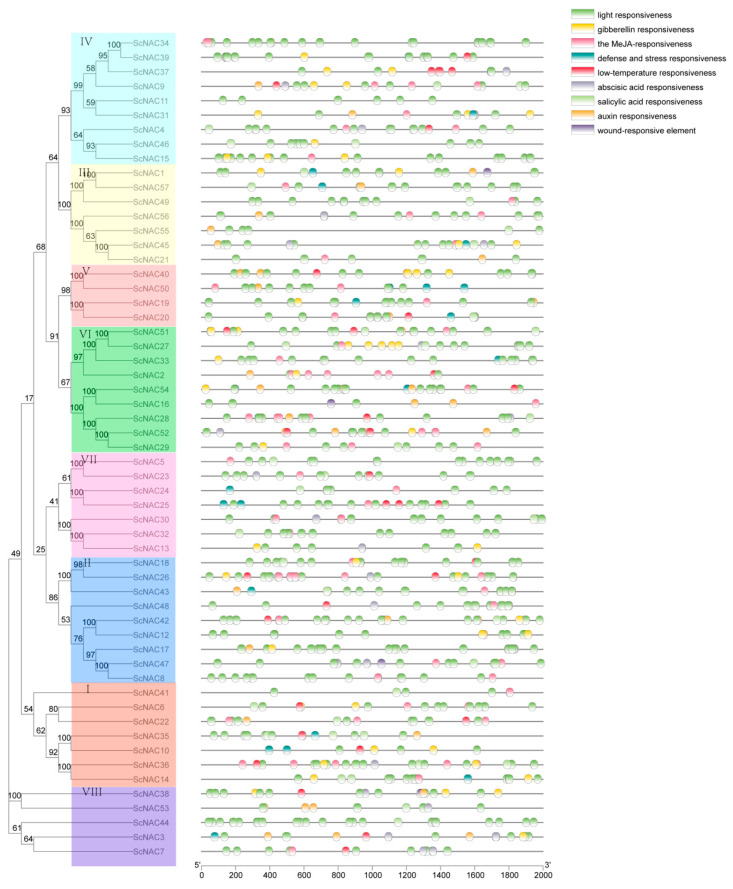
*Cis*-acting elements identified in 2000 bp region upstream of the Jojoba ScNAC promoter. Different *cis*-acting elements are represented by different colors, and some *cis*-acting elements overlap. NAC members were divided into groups I~VIII according to the phylogenetic analysis and marked with different colors.

*Cis*-acting elements are specific DNA sequences that have transcriptional regulatory functions in the same DNA molecule. In order to further understand the regulatory mechanism of NAC transcription family in the growth and development of Jojoba seeds, we extracted upstream 2000 bp sequences of 57 ScNAC genes from transcription start sites, and identified nine *cis*-acting elements using PlantCARE (Table S2). The ScNAC promoter contains a number of hormone-associated *cis*-acting elements, including gibberellin responsiveness, the MeJA-responsiveness, abscisic acid responsiveness, salicylic acid responsiveness, and auxin responsiveness (Figure 3). Some promoters contained stress-related elements, defense and stress responsiveness in 20 genes, low-temperature responsiveness in 25 genes, and wound-responsive elements in 4 genes. In addition, light responsiveness is present in all genes. These results suggest that NAC genes in Jojoba function and expression are regulated by the *cis*-elements involved in plant growth and development and stress.

### 3.5. Syntenic and Evolutionary Patterns of NAC TFs

**Figure 4:**
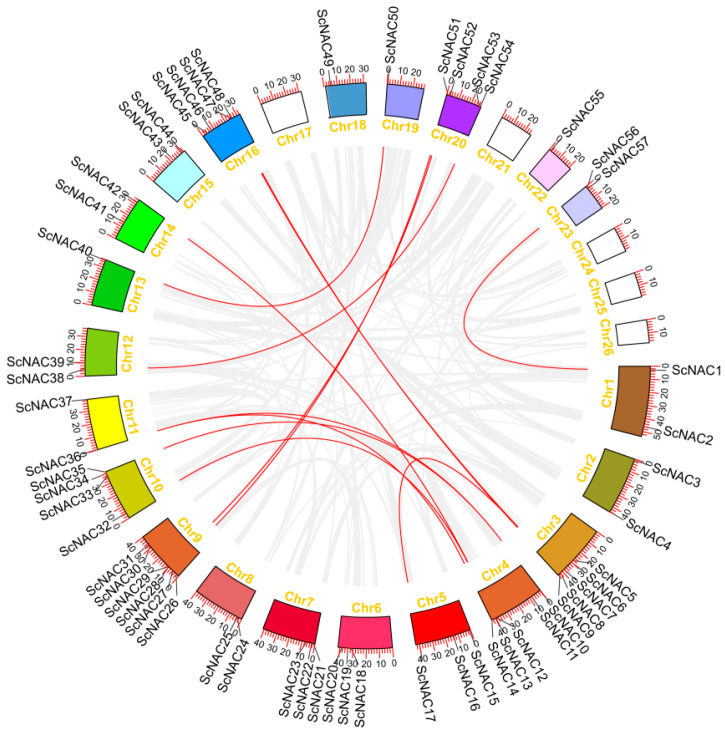
The location and relationship of ScNACs on chromosomes. White squares indicate no NAC genes distributed in that chromosome. Gray lines indicate all synteny regions in the Jojoba. Red lines indicate segmental duplicates of NAC gene pairs.

**Figure 5:**
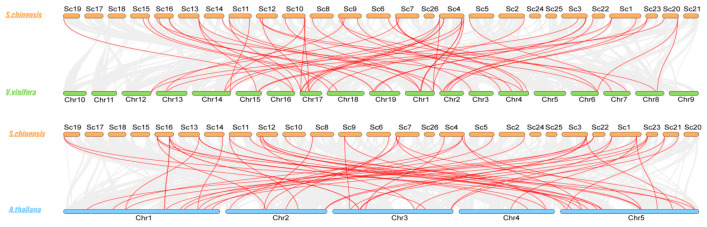
Synteny analysis of *S. chinensis* between *V. vinifera* and *A. thaliana*, respectively. Gray lines indicate regions of collinearity between Jojoba and other plants. Red lines indicate syntenic NAC gene pairs. The chromosomes of *S. chinensis*, *V. vinifera*, and *A. thaliana* are shown in orange, green, and blue, respectively.

Gene replication events exist widely in all species and provide the necessary materials for species evolution [43]. To further investigate the evolution of NAC TFs in Jojoba, we explored genome duplication events by syntenic analysis. In total, 12 gene pairs appeared to originate from segmental duplication, and 24 (42%) ScNACs were duplicated due to whole-genome duplication (WGD) (Figure 4). Previous studies have suggested that Jojoba diverged from *V. vinifera* about 100 million years ago [25]. We performed collinearity analysis between Jojoba and two representative plants: Arabidopsis and grape. The Jojoba NAC genes had 31 and 41 homologous pairs with Arabidopsis and grape, respectively (Figure 5).

To estimate the rate of evolution and selection pressure in Jojoba, we calculated the nonsynonymous substitution rate (Ka), synonymous substitution rate (Ks), and Ka/Ks for 10 pairs of homologous ScNAC. The Ks value of ScNAC ranged from 0.8458 to 2.8582, and the divergence time was 60.4–204.2 MAY. The Ka/Ks values of gene pairs in ScNAC were all less than 1.0, indicating that these genes may have undergone purifying selection during evolution.

### 3.6. Expression Profiles of ScNAC Based on RNA-Seq

To further investigate the function of ScNACs during the development of Jojoba seeds, we analyzed ScNACs expression in different tissues (i) and stages (ii) of developing seeds based on published gene expression data. The liquid wax esters (WEs) and triacylglycerols (TAGs) are the two major lipid storage substances in Jojoba seeds. Based on a previous study [25], WE and TAG were differentially enriched in seed tissues, WE mainly existed in cotyledons, and TAG mainly existed in the embryonic axis. We also analyzed the differential expression of ScNACs gene in the cotyledon and embryonic axis tissues (iii) based on RNA-seq data.

**Figure 6:**
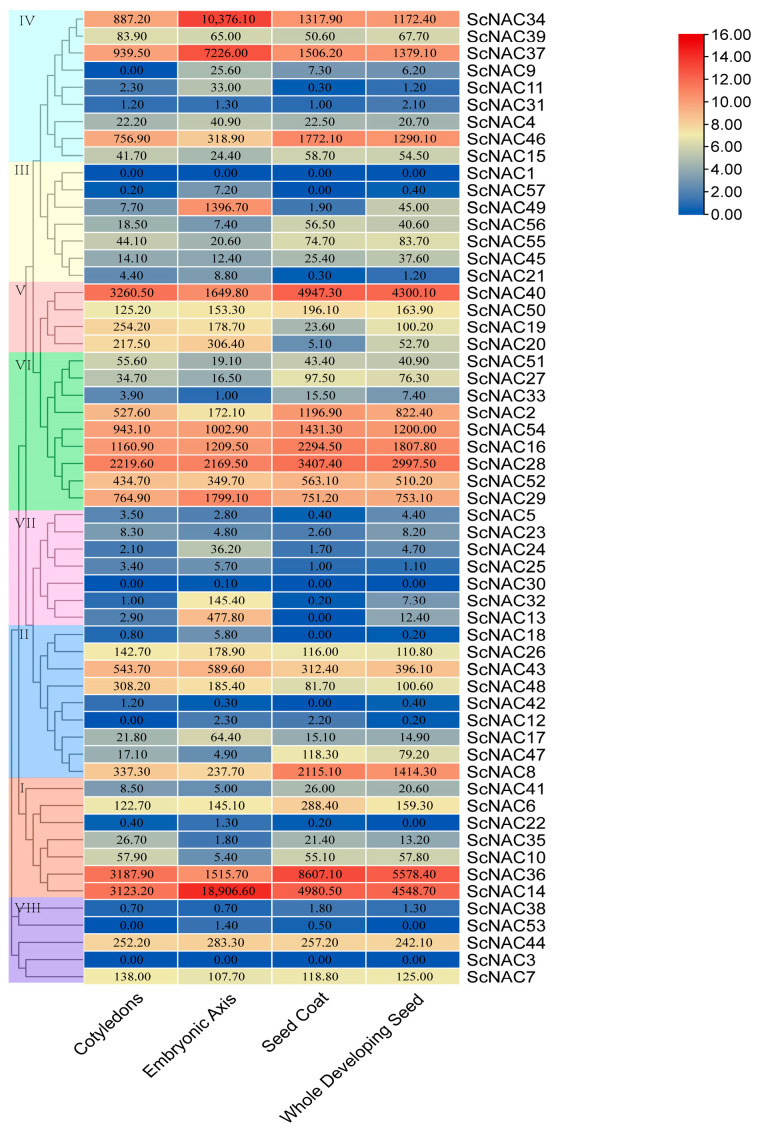
Heatmap of ScNAC expression in different seed tissues. NAC members were divided into groups I~VIII according to the phylogenetic analysis and marked with different colors. Samples were obtained from cotyledons, embryonic axis, seed coats, and whole seeds. Expression values were shown on color blocks. The color scale on the right indicates log_2_ expression of the mean value of ScNAC gene expression, with red representing high and blue representing low.

**Figure 7:**
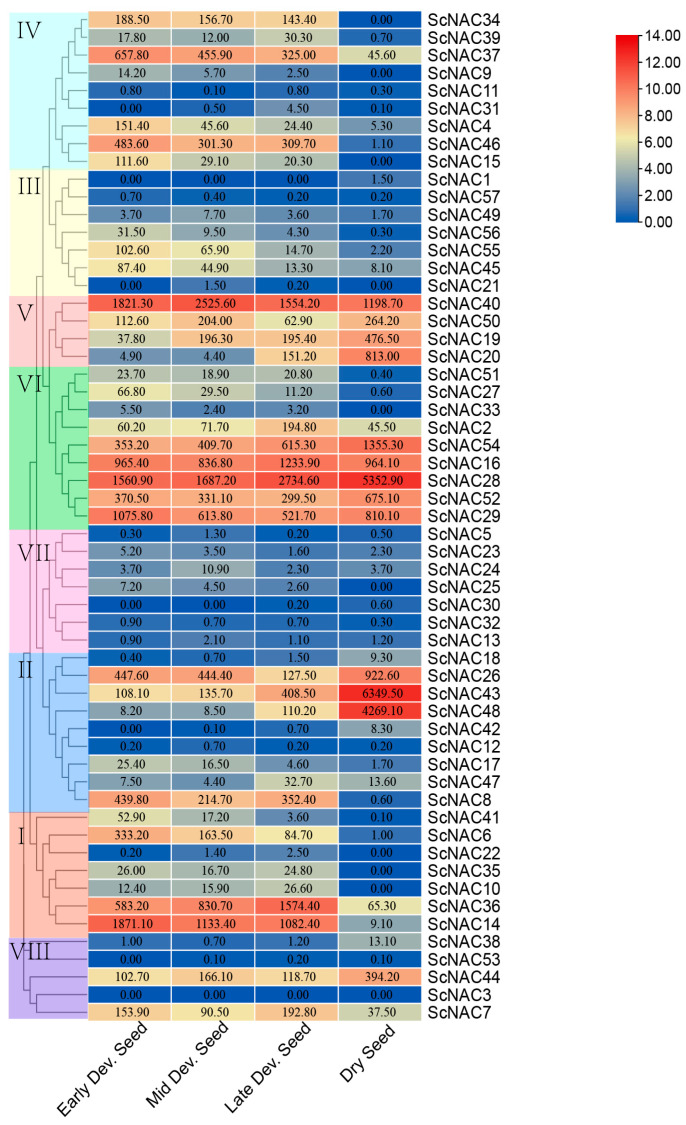
Heatmap of ScNAC expression at different stages of developing seeds. NAC members were divided into groups I~VIII according to the phylogenetic analysis and marked with different colors. Samples were obtained from early developing seeds (Early Dev. Seed), middle developing seeds (Mid Dev. Seed), late developing seeds (Late Dev. Seed), and mature dry seeds (Dry Seed). Expression values were shown on color blocks. The color scale on the right indicates log_2_ expression of the mean value of ScNAC gene expression, with red representing high and blue representing low.

**Figure 8:**
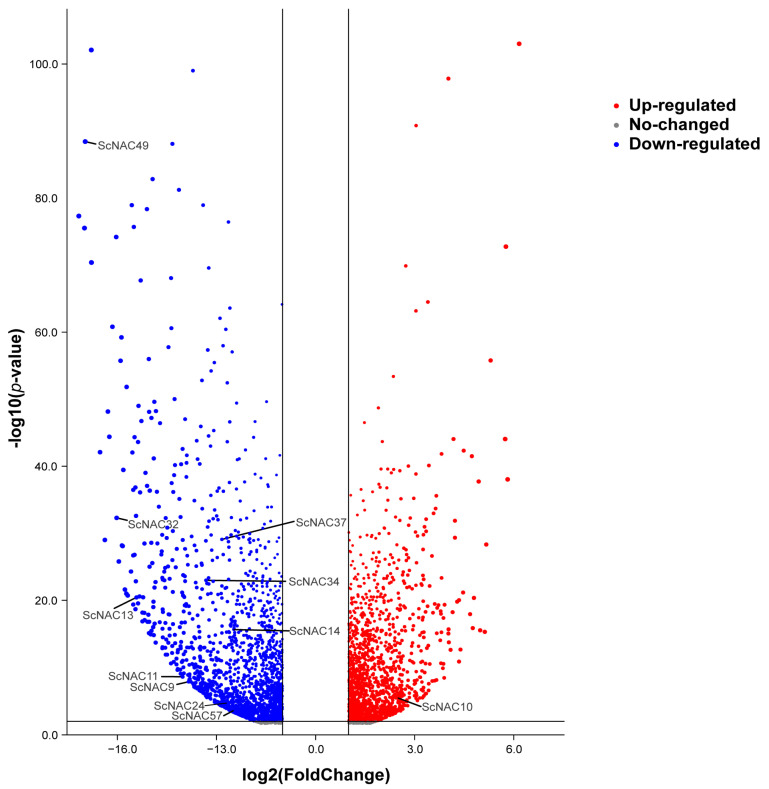
Differential expression of Jojoba genes in the cotyledon and embryonic axis tissues. Volcano plots were built based on significantly adjusted *p*-values less than 0.05 and absolute log_2_FC (log of fold change) values greater than 1. Genes whose expression is up-regulated are marked in red and genes whose expression is down-regulated are marked in blue. The NAC family members with an absolute value of log_2_FC greater than 2 are annotated.

(i) NAC gene family expression was analyzed in different seed tissues using published RNA-seq data (Figure 6). Among them, 46 genes were expressed in all tissues of the seed, 8 genes were expressed in some tissues, and 3 genes were not found to be expressed in the seed. Of the 46 genes that were all expressed in seed tissues, each gene was expressed at different levels in different seed tissues. The members from groups V and VI were expressed in all tissues, whereas the members from group VII were not expressed or expressed at very low levels in seeds. All members in V contained the *cis*-acting elements involved in light responsiveness. In addition, the intron/exon structures located in the V group are roughly the same in number and distribution. All members in V contained the *cis*-acting elements involved in the MeJA-responsiveness. This may indicate similar functions of ScNAC members in the same group during seed development. It is worth noting that the expression of three genes (*ScNAC34*, *ScNAC37*, *ScNAC14*) in the embryonic axis was much higher than that of other genes. The number and publication of exons/introns of these three genes were similar. Among them, the homologs of ScNAC34 and ScNAC37 in Arabidopsis belong to the same subfamily. They all had *cis*-acting elements involved in light response. This may indicate that these three genes (*ScNAC34*, *ScNAC37*, *ScNAC14*) play a critical role in Jojoba seed development. Similarly, *ClNAC68*, which is highly expressed in watermelon flesh, affects seed development cumulatively by controlling sugar and IAA [44]. *OsNAC129* is highly expressed in rice seeds and plays an important role in seed development [45].

(ii) NAC gene family expression was analyzed at different developmental stages using published RNA-seq data (Figure 7). Most ScNACs were expressed at different stages of seed development, but the level of gene expression is different. In total, 51 genes were expressed at all developmental stages (early, middle, late), of which 27 genes showed increased expression levels and 7 genes exhibited reduced expression levels in each developmental stage compared to the control group (mature dry seeds). In addition, 19 genes showed decreased expression levels with seed development. The expression levels of seven genes increased with seed development. Particularly, four of these genes (*ScNAC54*, *ScNAC28*, *ScNAC43*, *ScNAC36*) have high expression levels and an increasing trend at different stages of seed development. Members from group IV, most of which showed a downward trend, may indicate that they have a similar negative role in seed development. However, some members from subfamilies V and VI have distinct expression profiles, suggesting that structurally similar NAC genes have different functions. In conclusion, a positive or negative correlation between NAC gene expression and seed development may indicate that the ScNAC is involved in regulating the growth and development of Jojoba seeds, although the methods of regulation are complex and diverse.

(iii) To further understand the function of NAC genes in Jojoba seed development, we analyzed the differential expression of NAC genes in the cotyledon and embryonic axis tissues using RNA-seq data (Figure 8). According to the published RNA-seq data mentioned above, we obtained differentially expressed genes in the seed cotyledon and embryonic axis tissues. All differentially expressed Jojoba genes were shown in the volcano plot, and ScNAC genes were labeled among them (absolute value of log_2_FC greater than 2). A total of 11 ScNACs genes showed significant differential gene expression. Among these genes, three genes (*ScNAC34*, *ScNAC37*, *ScNAC39*) were highly expressed in both the cotyledon and embryonic axis tissues. Ten of these genes (*ScNAC49*, *ScNAC32*, *ScNAC37*, *ScNAC34*, *ScNAC13*, *ScNAC14*, *ScNAC11*, *ScNAC9*, *ScNAC24*, *ScNAC57*) were significantly down-regulated in cotyledons (log_2_FC < −2), and only one gene (*ScNAC10*) was significantly up-regulated (log_2_FC > 2). ScNACs were significantly more expressed in the embryonic axis than in the cotyledon, which may indicate ScNACs play an important regulatory role in the development of the embryonic axis.

## 4. Discussion

*Simmondsia chinensis* is an oil crop of important economic value in medicine and industry [24]. However, studies on its transcription factors have not been reported. The NAC gene family, as one of the widespread transcription families in plants, plays an important role in plant growth and development as well as in biological and abiotic stress. At present, the completion of the sequencing of the Jojoba genome makes it possible to explore the characteristics and functions of its important gene families. In this study, we identified 57 members of the NAC family from Jojoba. Based on previous studies between *Oryza sativa* and *Arabidopsis thaliana*, we divided the Jojoba and Arabidopsis sequences into 13 groups. The results showed that the distribution of the NAC gene family was not uniform among different subgroups. Subgroups A1, A2, A3, and A5 contained only NAC members from Arabidopsis, while the NAC members from Jojoba in B2 and B3 are more than Arabidopsis. This may be because Jojoba and Arabidopsis evolved in different environments, with the different differentiation of NAC gene families leading to inconsistent numbers of members in different subgroups.

The analysis of gene structure showed that there were differences in gene structure in the same group, such as the number of introns in V ranging from 1 to 4. This may be due to the insertion or splicing of gene fragments during evolution [46]. However, a similar gene structure and conserved motif distribution in the same group indicate that the members of the same group have similar functions. *Cis*-acting elements in promoters play an important role in regulating gene expression. In this study, we identified nine *cis*-acting elements that are involved in light response, hormonal response, and stress response. It has been reported that ABA plays a role in the regulation of seed development. ABA induced the expression of DELAY OF GERMINATION 1-LIKE 4 (DOGL4), which is a key factor mediating the increase of seed storage, including lipids, protein, and polysaccharides [47]. In addition, the ABA response plays a dominant role in the regulation of strawberry fruit ripening [48]. There are 42 *cis*-acting elements associated with the ABA response in ScNACs. In Arabidopsis thaliana, auxin response factor 2 works with SEEDSTICK (STK) and GODITA (GOA) to regulate the seed growth by promoting cell expansion in the seed coat [49]. In addition, 30 ScNAC genes have auxin response elements. The NAC family in Jojoba may play an important role in seed development.

Gene duplication is of great importance to species evolution, and the common modes of gene duplication in plant gene families are segmental and a tandem duplication [50]. Jojoba contained 57 NAC genes, 17 and 60 fewer than *V. vinifera* (74) [39] and Arabidopsis (117) [6], and 0.35, 0.46, and 0.56 times more than *Populus trichocarpa* (163) [7], *Phyllostachys edulis* (125) [8], and *Theobroma cacao* (102) [51], respectively. The number of NAC genes was significantly lower in Jojoba compared to other species, consistent with previous reports of WGD in Jojoba [25]. Therefore, we performed synteny analyses of NAC within and across species. The Jojoba NAC TFs have 24 genes derived from segmental duplication and only one from tandem duplication, and segmental duplication is the main source of amplification of the Jojoba NAC TFs. The synteny analysis of the Jojoba genome and the two other sequenced plant genomes showed that the collinearity between Jojoba and *V. vinifera* was more significant than that between Jojoba and Arabidopsis. This may be due to the additional crucifer genome duplication experienced by Arabidopsis compared to Jojoba and grape.

In order to explore the important role of the NAC family in developing Jojoba seeds, we analyzed the expression of ScNACs in different tissues and stages of developing seeds. The four genes (*ScNAC54*, *ScNAC28*, *ScNAC43*, *ScNAC36*) were highly expressed at different stages of seed development, and expression increased gradually with seed development. In *Arabidopsis Thaliana*, NAC103 positively regulates the ABA response during seed germination [52]. The NAC gene GmNAC42-1 in soybean (*Glycine max* L. *Merr*) is an important positive regulator of glyceollin biosynthesis [53]. This may indicate that these genes play a key positive role in the regulation of seed growth and development. Differential gene expression showed that ScNAC expression was significantly up-regulated in the embryonic axis. As the region of TAG enrichment, ScNACs may directly or indirectly participate in the regulation of TAG synthesis. Similarly, in oil palm (*Elaeis guineensis* Jacq.), NAC TFs are indirectly involved in fatty acid synthesis and lipid metabolism [54]. The combination of NAC transcription factor (*HvNAM1*) and RNA binding protein (*HvGR-RBP1*) extended grain fill duration, grain size, and protein concentration [55]. However, more genetic evidence is required to further understand the specific function of the NAC TFs during Jojoba seed development.

## 5. Conclusions

In this study, we performed the first genome-wide identification and analysis of the NAC TFs in Jojoba. We identified a total of 57 ScNACs belonging to eight different groups. Moreover, the structural and conserved motif analysis of NAC TFs indicated that they may be involved in light response, hormonal response, and stress response. Evolutionary analyses support that Jojoba experienced only one whole-genome triplication shared among all eudicots. Based on RNA-seq data, the specificity of ScNAC expression in seeds at different developmental stages and tissues was revealed. The seven genes (*ScNAC54*, *ScNAC28*, *ScNAC43*, *ScNAC36*, *ScNAC34*, *ScNAC37*, *ScNAC39*) may be closely related to seed development, and, of them, three genes (*ScNAC34*, *ScNAC37*, *ScNAC39*) may play a key role in the embryonic axis of developing seeds. In conclusion, this study has obtained more information on NAC TFs in Jojoba and laid the foundation for further studies on the specific functions of ScNACs in Jojoba seeds.

## Data Availability

All the data relevant to the study are included in the article or uploaded as Supplementary Materials.

## Supplementary Materials

The following supporting information can be downloaded at: https://www.mdpi.com/article/10.3390/cimb45070344/s1.
